# Application of Central Composite Design and Superimposition Approach for Optimization of Drying Parameters of Pretreated Cassava Flour

**DOI:** 10.3390/foods12112101

**Published:** 2023-05-23

**Authors:** Ellyas Alga Nainggolan, Jan Banout, Klara Urbanova

**Affiliations:** 1 Department of Sustainable Technologies, Faculty of Tropical AgriSciences, Czech University of Life Sciences Prague, Kamýcká 129, 16500 Prague, Czech Republic; 2 Department of Bioprocess Engineering, Faculty of Biotechnology, Institut Teknologi Del, Jl. Sisingamangaraja, Sitoluama, Laguboti, Toba 22381, North Sumatera, Indonesia

**Keywords:** blanching, cassava flour, central composite design, soaking, superimposition

## Abstract

The primary goals of this study were to identify the influence of temperature and drying time on pretreated cassava flour, as well as the optimal settings for the factors and to analyze the microstructure of cassava flour. The experiment was designed using the response surface methodology with central composite design and the superimposition approach in order to assess the effect of drying temperature (45.85–74.14 °C) and drying time (3.96–11.03 h) and the optimal drying conditions of the cassava flour investigated. Soaking and blanching were applied as pretreatments to freshly sliced cassava tubers. The value moisture content of cassava flour was between 6.22% and 11.07%, whereas the observed whiteness index in cassava flour ranged from 72.62 to 92.67 in all pretreated cassava flour samples. Through analysis of variance, each drying factor, their interaction, and all squared terms had a substantial impact on moisture content and whiteness index. The optimized values for drying temperature and drying time for each pretreated cassava flour were 70 °C and 10 h, respectively. The microstructure showed a non-gelatinized, relatively homogeneous in size and shape sample with pretreatment soaked in distilled water at room temperature. These study results are relevant to the development of more sustainable cassava flour production.

## 1. Introduction

Indonesia is an agrarian nation where the agricultural sector is one of the primary economic development drivers. In Indonesia, numerous agricultural crops are cultivated, including tubers, cereals, legumes, vegetables, and fruits. In 2020, 18.5 million tons of cassava tubers (*Manihot esculenta* Crantz) were the most produced source of carbohydrates other than rice [1]. Indonesia is one of the six largest producers of cassava in the world, along with Nigeria, Democratic Republic of the Congo, Thailand, Ghana, and Brazil [2]. Cassava tubers are the most commonly consumed component of the cassava plant; this part is rich in starch and is the primary storage organ in cassava plants [3,4]. Cassava tubers are one of the most promising agricultural products for diversification into several food varieties. In Indonesia, cassava tubers are processed into a variety of products, including tapioca, mocaf, cassava flour, tapai, chips, and tiwul. Cassava flour is produced from cassava tubers that have been processed using an uncomplicated drying technique [5].

In an effort to expand the use of cassava as a food, postharvest handling and flour processing are employed. Compared to fresh cassava tubers, cassava flour has a longer shelf life and a larger range of applications. The appearance of vascular streaks with bluish-black staining is a sign of postharvest physiological decline in cassava tubers. Microbial activity is the primary cause of cassava tuber destruction [6,7]. Physical (blanching) and chemical (calcium chloride, citric acid, and ascorbic acid) pretreatments are frequently used to prevent the browning and discoloration of tubers caused by enzymatic action. Some of the literature describes the use of blanching as well as ascorbic acid, sulfite, and citric acid in the production of yam flour [8,9]; the use of sulfite in the production of sweet potato starch [10,11]; and the use of calcium chloride treatment in the production of cassava chips [12,13]. However, there are just a few reports using blanching and soaking as the only pretreatments for cassava flour processing.

In recent years, one-factor-at-a-time (OFAT) analysis has been used extensively in the food processing literature, particularly for drying technologies. Statistical analysis and regression coefficient models or the mathematical models are required to predict the process conditions for drying cassava flour. Design of experiments (DOE) provides a number of advantages over conventional analysis, including minimal resource requirements (number of trials, time, materials, and labor), exact prediction findings on the major elements and their interactions, and the capacity to study a large number of factors [14]. Response surface methodology (RSM) is a statistical technique used to determine the relationship between response variables and a set of input variables [15]. RSM is a statistical and mathematical technique that can be utilized to create, develop, and optimize processes, formulations, or even both [16]. RSM is currently one of the most often used optimization techniques in the world of food technology and engineering. RSM has been used for process optimization in several studies: 1. determine the drying behavior of cassava chips at various temperatures using different cutting shapes [17]; 2. impact of temperature and drying time on the thermal and physical characteristics of cassava flour [18]; 3. as a tool to discover the interactive impact of pretreatment and drying process on the physicochemical of cassava flour [19]; 4. optimization of drying parameters for convective drying and drum drying of sweet potatoes [20,21].

There is currently a lack of information regarding the influence of blanching and soaking on cassava flour when the drying process (temperature and time) is optimized using RSM, particularly the central composite design (CCD) and superimposition approach. As pretreatments in this investigation, soaking in distilled water and blanching were applied separately. The objectives of this study were to: (i) investigate the effect of drying parameters on the moisture content (*MC*) and whiteness index (*WI*) of cassava flour; (ii) carry out optimization, verification, and superimposition processes to achieve the optimal combination of factors that generate minimum *MC* and maximum *WI* of cassava flour; and (iii) analyze the microstructure of cassava flour and evaluate the results.

## 2. Materials and Methods

### 2.1. Design of Experiment Based on RSM

The software Design Expert version 13.0.5.0 (Stat-Ease Inc., Minneapolis, MN, USA) was used to construct an experimental matrix for processing samples of cassava flour. When designing experiments with RSM, there were two drying parameters that served as the basis: the drying temperature (*T*_1_) and the drying time (*T*_2_). The three pretreatments were applied independently, and then each treatment was processed with the *T*_1_ and *T*_2_ configurations according to the experimental matrix. As for the responses of the two factors, which are the moisture content (*MC*) and whiteness index (*WI*) of cassava flour. Table 1 shows the five specified levels and operating ranges for the CCD.

### 2.2. Experiment Design

Based on the five levels, two factors, and three replications applied to all design points, the CCD developed by Design Expert software (Stat-Ease Inc., Minneapolis, MN, USA) generated a total of 39 experiments. These variables were chosen because they have a considerable impact on the responses and the permissible working range, as documented in the literature. Table 2 displays the full CCD, including both coded and uncoded factor values. The total value of the block is 1 and the experiments are conducted in a random order.

The significance of the main components and their interactions was determined using an analysis of variance (ANOVA) with a significance threshold of 95% and a *p*-value of 0.050. The mathematical models were derived from the ANOVA table. These models were then used for optimization purposes, the outcome of which was determined by the value of the correlation coefficient, *R*^2^. The experimental data were fitted to a second-order polynomial model to generate a regression coefficient model. Equation (1) illustrates the model form for response surface analysis:

(1) $$Y=\beta_{0}+\overset{3}{\underset{t=1}{\sum }}\beta_{i}\;X_{i}+\overset{3}{\underset{i}{\sum }}\beta_{ii}X_{i}^{2}+\overset{2}{\underset{i-1}{\sum }}\;\overset{3}{\underset{j=i+1}{\sum }}\beta_{ij}\;X_{i}X_{j}$$

where *Y* is the response, *β*_0_*, β_i_, β_ii_*, and *β_ij_* are the regression coefficients for the intercept, linear, quadratic, and interaction, respectively. *X_i_* and *X_j_* are coded values in independent variables [22].

### 2.3. Raw Materials

The tubers of cassava were purchased in a local market in the village of Pasar Laguboti, which is located in the Laguboti District of the Toba Regency in the province of North Sumatra, Indonesia. The local farmers in the village harvested cassava tubers 13 to 17 months after planting. The cassava tubers were sorted before being cleaned in order to eliminate soil and prevent contamination during processing. To minimize injury to the tubers, processing occurs only after 24 h have passed since their collection [13].

### 2.4. Processing of Pretreated Cassava Flour

The procedure described by the Indonesian Agency for Agricultural Research and Development [23] is modified for the processing of cassava flour. The modification of the procedure includes pretreatments consisting of blanching and soaking each experimental sample in distilled water. After cleaning the cassava tubers, they were manually peeled and sliced into 3 × 3 × 1 ± 1 cm (length × width × thickness) pieces. Freshly sliced cassava tubers were subjected to three pretreatments: A (blanched at 80 ± 2 °C for 5 min then soaked in distilled water for 48 h), B (soaked in distilled water for 48 h then blanched at 80 ± 2 °C for 5 min), and C (soaked in distilled water for 72 h at room temperature, 24 ± 4 °C). The cassava slices were then dried in a drying machine (400 W Food Dehydrator, ATHOME collection, West Jakarta, Indonesia) according to the experimental matrix at the temperature and time stated (Table 2). The parameters for drying in this study were drying temperature (45.85–74.14 °C) and drying time (3.96–11.03 h). A dry milling machine (HR 2115 Dry Mill Blender, PT. Philips Batam, Batam, Indonesia) was utilized to process the dry chips. The flour obtained from the mill was sieved and kept at room temperature in a plastic sample bag until further analysis.

### 2.5. MC Analysis

The *MC* of cassava flour was calculated using standard analytical chemistry procedures [24]. The percentage of *MC* is expressed on a dry basis using the following Equation (2):

(2) $$MC\;\left(\%\right)=\frac{W_{t}\;\left(g\right)}{W_{i}\;\left(g\right)}$$

where, *MC* is moisture content; *W_t_* is the weight of the sample at time *t*; and *W_i_* is the initial weight of sample.

### 2.6. Color Measurement

Using a colorimeter (CS-10, Hangzhou Caipu Technology Co., Ltd., Hangzhou, China), samples of cassava flour were measured in three repetitions. The instrument was calibrated using a bright white standard reference tile and a bright black standard reference tile. During color assessment, L* (brightness), a* (positive values indicate redness and negative values indicate greenness), and b* (positive values represent yellowness and negative values represent blueness) values were collected. According to Torbica et al. [25], the value of the *WI* can be quantitatively determined by combining the L*, a*, and b* components into a single computed term. The formula for *WI* can be found as follows:

(3) $$WI=100-\sqrt{a^{*2}+b^{*2}+{\left(100-L^{*}\right)}^{2}\;}$$

### 2.7. Microstructure Analysis

Utilizing a scanning electron microscope (SEM) (EVO MA10, Carl Zeiss Pvt. Ltd., Oberkochen, Germany), morphological structural analysis was performed with the purpose of determining the effect of pretreatments (A, B, and C) and drying parameters (*T*_1_ and *T*_2_) on the structures of cassava flour particles. Double-sided tape was used to adhere the samples to the bronze visualization portions. A thin layer of gold was coated on the surface of the sample using a sputter period of 60 s and a sputter power of 20 mA. Surface pictures were captured using an SE (secondary electron) detector with a working distance (WD) of 11.5–12 mm and an extra-high-tension (EHT) of 11.0 kV at 1000× magnification for all samples.

## 3. Results and Discussion

Table 3 displays the design configuration derived from the Design Expert program as well as the experimental responses data (*MC* and *WI*). Temperature and drying time are two experimental design variables represented by *T*_1_ and *T*_2_, respectively.

### 3.1. Statistical Analysis of MC

According to the results of the ANOVA shown in Table 4, all of the primary factors (*T*_1_ and *T*_2_) are highly significant at a *p*-value of 0.000. The coefficients of determination (*R*^2^) of the samples with pretreatments A, B, and C, respectively, are 0.9624, 0.9713, and 0.9648. They indicate that the *MC* in each sample A, B, and C is correlated to *T*_1_ and *T*_2_ by 96.24%, 97.13%, and 96.48%, respectively. If *R*^2^ equals 1, it indicates that the regression coefficient model can predict the optimal value with a high degree of accuracy. The *p*-value obtained for the lack of fit test was not statistically significant for all pretreatment samples. The high value of the regression and the statistically insignificant lack of fit indicate that the model fits the data well when it is applied.

Factor interactions (*T*_1_*T_2_) and all squared terms (*T*_1_*T_1_ and *T*_2_*T_2_) are statistically significant at a *p*-value less than 0.050. Due to the largest absolute coefficient value, primary factors (*T*_1_ and *T*_2_) are seen to have the highest impact on the response for all sample pretreatments. The significant (*p*-value 0.000) squared term indicates that the interaction between factors and responses follows a curved line. The Equations (4)–(6) present the regression coefficient model of pretreatments *A*, *B*, and *C*, respectively, for the several variables that contribute to the *MC* of cassava flour:

(4) $$Y_{MC}=11.0887-0.7618\left(T_{1}\right)-0.7415\left(T_{2}\right)+0.5230{\left(T_{1}\right)}^{2}+0.4805{\left(T_{2}\right)}^{2}-0.5250\left(T_{1}\right)\left(T_{2}\right)$$

(5) $$Y_{MC}=11.6787-0.7562\left(T_{1}\right)+0.9402\left(T_{2}\right)+0.5959{\left(T_{1}\right)}^{2}+0.6559{\left(T_{2}\right)}^{2}-0.6708\left(T_{1}\right)\left(T_{2}\right)\;$$

(6) $$Y_{MC}=7.7473-0.7878\left(T_{1}\right)+0.8175\left(T_{2}\right)+0.4430{\left(T_{1}\right)}^{2}+0.4938{\left(T_{2}\right)}^{2}-0.6725\left(T_{1}\right)\left(T_{2}\right)\;$$

where *Y_MC_* represents *MC* as the response, whereas *T*_1_ and *T*_2_ are the temperature and drying time, respectively. This mathematical model can be used to determine and assess the impact of variables on the *MC* of cassava flour.

### 3.2. Effect of Factors on MC

**Figure 1:**
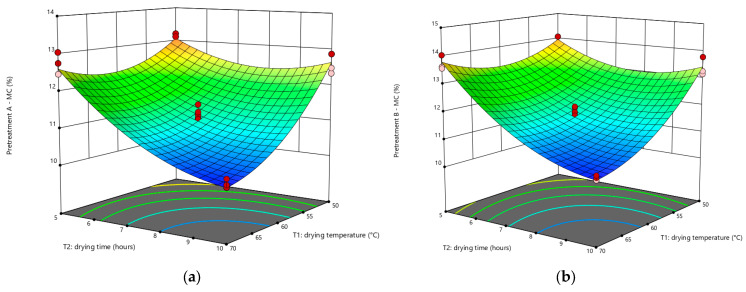

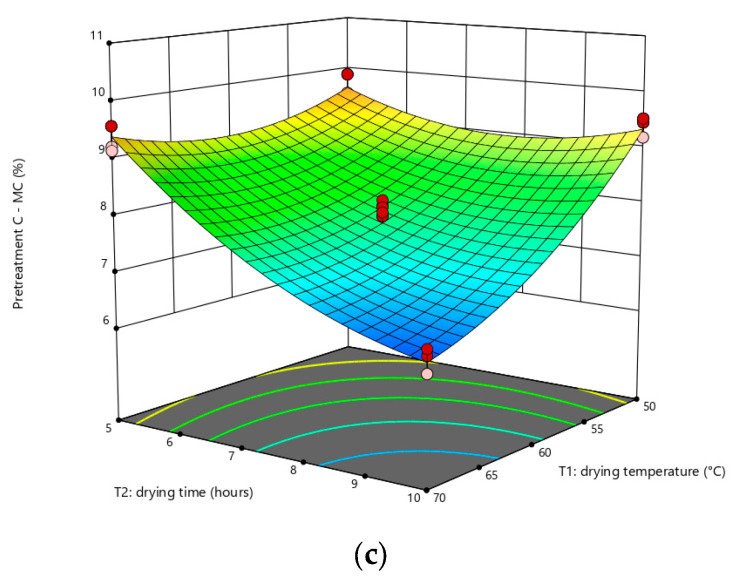
Response surface plot for *MC* of cassava flour with pretreatment (**a**) A; (**b**) B; (**c**) C. The blue, green, yellow, and red colors on the surface represent the gradient range from the lowest to the greatest response value, respectively. The red dot represents the response value above the surface, while the pink dot represents the response value below the surface.

The impact of *T*_1_ and *T*_2_ on the *MC* of cassava flour was determined using ANOVA and regression coefficient models based on statistical analysis. Figure 1 illustrates the effect of temperature and drying time on the *MC* of cassava flour with a 3D surface graph. Drying conditions with low MC were detected at drying temperatures of 70 °C for 10 h for all pretreated samples. The lowest observed concentration of MC in cassava flour treated with C was 6.22%. Temperature and time are among the most critical elements that directly influence the drying kinetics during thermal drying.

Blanching is accomplished by applying an instant and modest thermal treatment to the sample. Enzymatic inactivation, physical structure alteration, and flavor and nutritional content preservation are all targets [26,27]. The serial soaking–blanching–boiling of cassava chips produced a higher drying rate and lower moisture desorption [27]. The *MC* of cassava flour ranged from 10.07% to 13.29% in samples with pretreatment A, between 11.07% and 14.07% in samples with pretreatment B, and between 6.22% and 10.13% in samples with pretreatment C. The *MC* of samples prepared with blanching was higher than that of samples not pretreated with blanching under the same drying conditions. This phenomenon arises due to the fact that blanching promotes starch gelatinization and that during the subsequent drying process, a barrier layer forms on the surface of the sample, which minimizes the amount of water that is transferred from the sample to the atmospheric air [28,29]. Ai et al. [30] also reported that higher heating slowed the drying process and lengthened the dehydration period. Similar findings were discovered by Chen et al. [31], who discovered that the *MC* in unblanched samples of yam flour was lower than blanched samples of the flour. They found that the water-binding capacity (WBC) value of the blanched samples was higher compared to the unblanched samples of yam flour. According to Tacer-Caba et al. [32], higher blanching temperatures and other thermal operations lead to a greater degree of starch gelatinization. The degree of gelatinization and starch fragmentation are the two most important factors influencing WBC [33].

**Figure 2:**
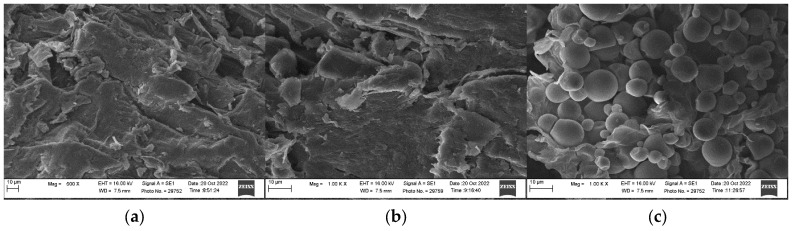
Microstructure of cassava flour with pretreatment: (**a**) A; (**b**) B; and (**c**) C at 1000× magnification after being dried at 70 °C for 10 h.

Figure 2 depicts a microscopic picture of the A, B, and C samples, which were processed at 70 °C for 10 h. Oval and spherical granules were observed in samples treated with C. The sample granules that followed the blanching procedure presented a variety of forms and sizes, with some of them having been gelatinized. The granules represented in Figure 2c are non-gelatinized and relatively homogeneous in size and shape. Figure 2a,b show some of the granules that have been gelatinized into enormous masses with block-like and irregular structures as well as voids and rough surfaces. These results are the consequence of the partial gelatinization and subsequent retrogradation of starch appearing to be held together by binding factors such as water and gelatinized starch [34,35].

### 3.3. Statistical Analysis of WI

As can be seen in Table 5 of the results of the analysis of variance (ANOVA), the findings revealed that all of the primary factors (*T*_1_ and *T*_2_) were extremely significant with a *p*-value of 0.000. The coefficients of determination of the samples with pretreatments A, B, and C, respectively, are 0.9774, 0.9772, and 0.9657. They indicate that the *WI* in each sample A, B, and C is correlated to *T*_1_ and *T*_2_ by 97.74%, 97.72%, and 96.57%, respectively. If the value of *R*^2^ is 1.0000, then this can be taken as the ability of the regression coefficient model to accurately predict the optimum value.

Factor interactions (*T*_1_*T_2_) and all squared components (*T*_1_*T_1_ and *T*_2_*T_2_) are statistically significant at a *p*-value less than 0.050. The squared factors (*T*_1_*T_1_ and *T*_2_*T_2_) had the most impact on the response, as indicated by the highest absolute coefficient value of 0.9171 to 1.4396. *T*_1_*T_2_ obtained a *p*-value of 0.003, 0.029, and 0.004, respectively, for the samples with pretreatment A, B, and C for the interaction between the two factors, indicating that there is a significant association between the two factors. The squared term reveals that the relationship between the factors and the responses forms a curved line, and its significance is demonstrated by the fact that the *p*-value is less than 0.050. The regression coefficient model for the parameters influencing the *WI* of cassava flour is shown in Equations (7)–(9) for the sample with pretreatments A, B, and C, respectively.

(7) $$Y_{WI}=80.8053+0.3702\left(T_{1}\right)-0.2683\left(T_{2}\right)+0.9646{\left(T_{1}\right)}^{2}+1.4096{\left(T_{2}\right)}^{2}+0.1875\left(T_{1}\right)\left(T_{2}\right)\;$$

(8) $$Y_{WI}=77.8273+0.4150\left(T_{1}\right)-0.2942\left(T_{2}\right)+1.0068{\left(T_{1}\right)}^{2}+1.4276{\left(T_{2}\right)}^{2}+0.1392\left(T_{1}\right)\left(T_{2}\right)$$

(9) $$Y_{WI}=88.79+0.3782\left(T_{1}\right)-0.2026\left(T_{2}\right)+0.9171{\left(T_{1}\right)}^{2}+1.4396{\left(T_{2}\right)}^{2}+0.2875\left(T_{1}\right)\left(T_{2}\right)$$

*Y_WI_* represents the response for *WI*, whereas *T*_1_ and *T*_2_ represent the temperature and drying time, respectively. Calculating and analyzing the influence of various factors on the *WI* of cassava flour is possible with the help of these regression coefficient models. The mathematical model demonstrates that the *p*-value of the lack of fit test and the regression value of the model are progressively high and insignificant. The non-significant lack of fit and high regression value indicate that the implemented model is well-fitting.

### 3.4. Effect of Factors on WI

In terms of customer preference for the physical quality of food, color is a crucial component, particularly with regard to flour-based products. Morrot et al. and Zellner & Durlach [36,37] reported that drying circumstances altered the color of various agricultural products. Temperature and drying time are responsible for the discoloration caused by thermal and oxidation reactions during drying [38,39,40].

Cassava flour with acceptable physical and color qualities is white flour. Akintunde and Tunde-Akintunde [41] similarly reported low a* values (−0.07–7.50) and b* values (4.92–8.99) and high L* values (52–80.02) for cassava starch and yam flour, which is consistent with the findings of this study. However, the modest variances in L*, a*, and b* values can be related to changes in the varieties that were utilized and the drying procedures that were used. *WI* reflects the degree of whiteness of food products and the extent of color transformation during food processing [42]. The analysis of the 3D surface graph depicting variations in *WI* angles under different drying conditions of flour indicates that cassava drying at the temperatures and time ranges used in this study can assist in preserving the color of cassava flour, thereby increasing consumer acceptance, utilization, and application in the food industry.

**Figure 3:**
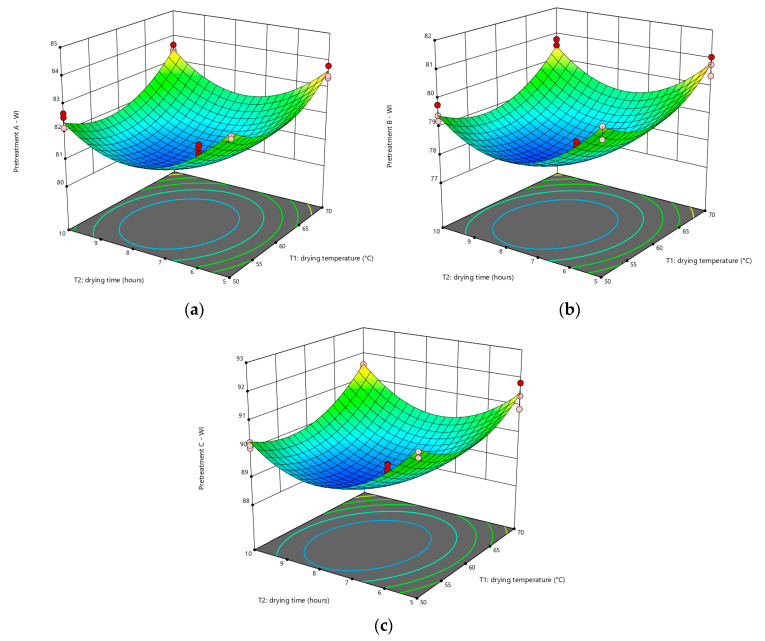
Response surface plot for *WI* of cassava flour with pretreatment (**a**) A; (**b**) B; (**c**) C. The blue, green, yellow, and red colors on the surface represent the gradient range from the lowest to the greatest response value, respectively. The red dot represents the response value above the surface, while the pink dot represents the response value below the surface.

**Figure 4:**
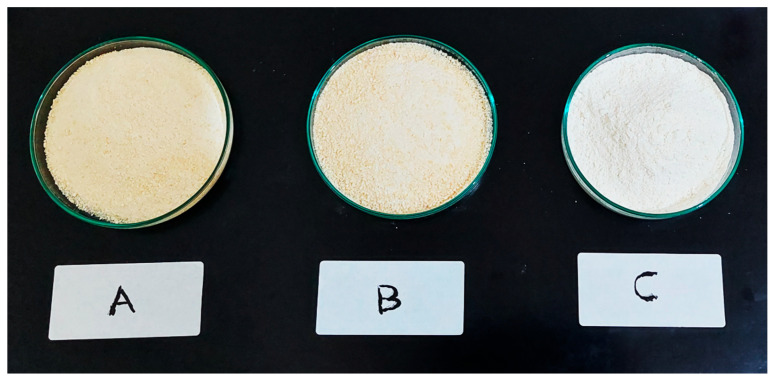
The pretreated cassava flour after being dried at 70 °C for 10 h.

Figure 3 depicts the 3D surface graphs illustrating the impact of *T*_1_ and *T*_2_ on *WI*. The *WI* of cassava flour ranged from 80.48 to 84.05 in samples with pretreatment A, between 77.62 and 81.27 in samples with pretreatment B, and between 88.56 and 92.07 in samples with pretreatment C. The highest *WI* values were found in samples pretreated with C that dried at 60 °C for 3.96 h. This could imply that blanching cassava tubers for 5 min at 80 ± 2 °C in hot water was sufficient to drive an increasing non-enzymatic browning reaction. Quayson et al. [43] reported that non-enzymatic browning intensities of yam decreased as soaking time increased. They also discovered that as blanching time increased, non-enzymatic browning levels increased. According to a study done by Sanful et al. [44], samples that were not pretreated showed higher L* values than those that had been blanched in yam flour. Figure 4 displays the cassava flour produced under drying conditions of 70 °C for 10 h. As seen in the picture, cassava flour treated with pretreatment C is whiter than cassava flour treated with pretreatments A and B. The photos represent the *WI* value, which indicates that cassava flour with pretreatment C has the highest *WI* value among the others.

### 3.5. Optimization of MC and WI

**Figure 5:**
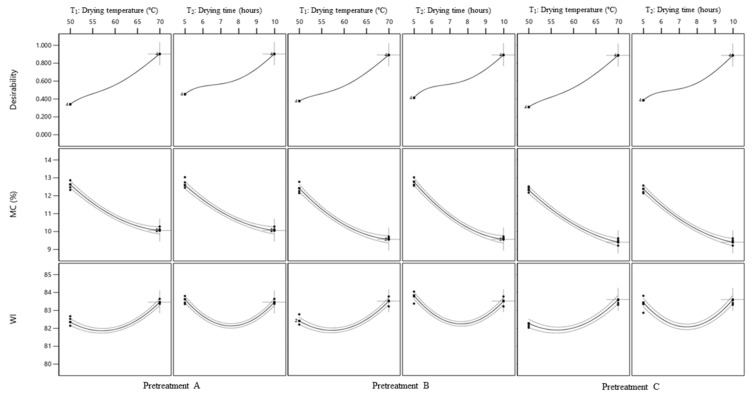
Optimization plot for the optimum *T*_1_ and *T*_2_.

The optimization process was conducted to determine the optimal temperature and drying time for producing cassava flour with the lowest *MC* and highest *WI* values. All factors were within the workable range because the desired composite value, *D*, was calculated to be close to 1. The *D* values of cassava flour with pretreatments A, B, and C, respectively, were 0.90, 0.89, and 0.89. Figure 5 displays the cassava flour optimization plot for all pretreated cassava flour. The optimal values for *T*_1_ and *T*_2_ for all pretreated cassava flour were 70 °C and 10 h, respectively. Cassava flour with pretreatment A had an *MC* of 10.06% and a *WI* of 83.47 in the optimum drying parameters, whereas cassava flour with pretreatment B had an *MC* of 10.63% and a *WI* of 80.52. Cassava flour with pretreatment C had the lowest *MC* (6.41%) and the highest *WI* (91.61) compared to the other pretreatments in the optimum drying conditions. These findings are consistent with those obtained in other investigations, which found a minimum *MC* and maximum *WI* in each type of processed cassava flour. Omolola et al. [18] reported that the *WI* and L* of the cassava flour samples were relatively high. Flour typically has an *MC* of less than 12% [45]. Furthermore, a low moisture content is required to limit microbial growth in food [46].

### 3.6. Experimental Verification

Experimental verification is the final phase in the modelling procedure and is used to check that the predicted model (the regression coefficient model) is accurate [47]. The experiment was conducted under optimal conditions derived from the optimization plot, with three replicates of each sample. According to the data presented in Table 6, the mean relative deviations for *MC* and *WI* were, respectively, 1.48% and 0.12% for samples that had been subjected to pretreatment A; 1.48% and 0.16% for samples that had been subjected to pretreatment B; and 1.29% and 0.16% for samples that had been subjected to pretreatment C. By comparing the experimental (actual) value to the predicted figures, this verifies the predictability of the model and indicates that the RSM-based empirical model can accurately explain the correlation between the variables and the goal response, thereby successfully confirming the optimal process conditions. The *MC* of cassava flour samples processed under varied drying validation conditions ranged from 7.43% to 10.50%, whereas *WI* values ranged from 80.38 to 91.83. According to Onitilo et al. [48], the percentage *MC* of cassava flour ranges from 3.59% to 11.53%, and these results fall within that range. Similarly, the *WI* follows the same pattern as the L* value. Omolola et al. [18] recorded cassava flour *WI* values between 82.88 and 89.42.

### 3.7. Contour Plots Superimposition

**Figure 6:**
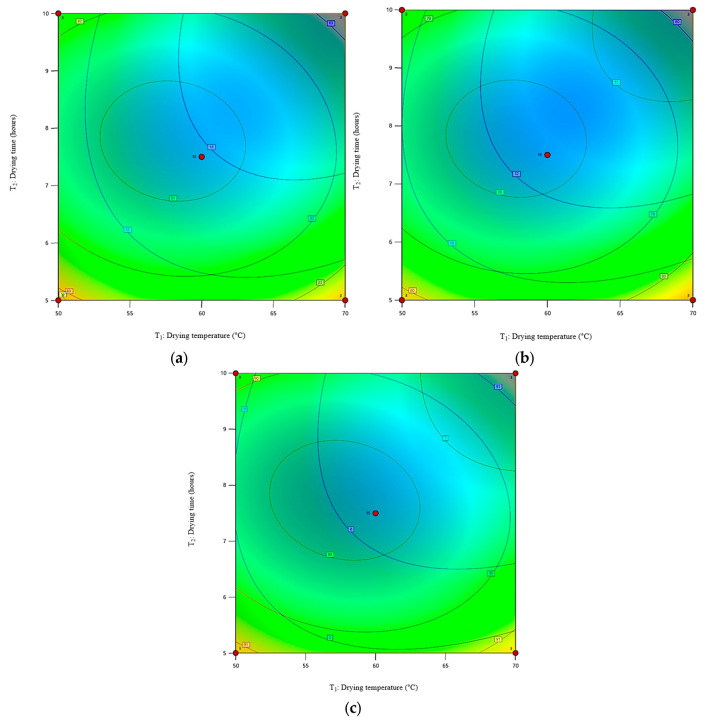
Superimposition of the contour plots for the optimum drying conditions for cassava flour with (**a**) A, (**b**) B, and (**c**) C pretreatments.

The superimposition of contour plots is the approach used to plot overlay graphs for diverse response surfaces. This technique is superior to the conventional OFAT approach, which does not account for the interaction between the selected variables and involves complex experiments [49]. The overlay contour plot functions as a convenient template for evaluating the response for every given factor value within the defined range. The optimal range of achievable drying settings for pretreating cassava flour is represented in Figure 6. Based on the contour plots that were superimposed, the ideal range for the minimum *MC* values and the maximum *WI* values was determined to be 70 °C and 10 h for all pretreatments. The grey areas represent the optimal drying area for all pretreated cassava flour samples.

## 4. Conclusions

The impact of temperature and drying time on the moisture content and whiteness index of each pretreated cassava flour has been examined. Temperature and drying time had a substantial impact on pretreated cassava flour’s *MC* and *WI*, as shown by statistical analysis utilizing RSM and CCD. In all experimental designs, the lowest *MC* of cassava flour was between 6.22% and 11.07%, whereas the greatest observed *WI* in cassava flour ranged from 72.62 to 92.67 in all pretreated cassava flour samples. The microstructure revealed that the highest *MC* sample featured starch gelatinization, and a barrier layer formed on the surface of the sample during the drying process. The thermal processing of cassava tubers led to a greater degree of starch gelatinization.

The constructed prediction models, or the regression coefficient models, proved to be highly accurate. The superimpositions of the contour plots were successfully expanded to pinpoint the optimum area of drying parameters for the minimum *MC* and maximum *WI* values, which were identified under process conditions of 70 °C and a drying duration of 10 h for all pretreated cassava flour samples. According to the validation results, the average relative deviation for the *MC* and *WI* ranged from 0.12% to 1.48%.

There are a number of possible research projects that have been explored, including the cassava flour drying kinetics model. Furthermore, studies on the interaction between pretreatment and drying conditions, in addition to other drying methods, have the potential to increase the quality of cassava flour.

## Figures and Tables

**Table 1:** Factors and levels used for *MC* and *WI* analysis.

Factor	Unit	Notation	Level				
			−1.414	−1	0	1	1.414
Temperature	°C	*T*_1_	45.8579	50	60	70	74.1421
Time	Hours	*T*_2_	3.9644	5	7.5	10	11.0355

**Table 2:** Design matrix of the experiment.

Sample			Coded Factor		Uncoded Factor	
Pretreatment A	Pretreatment B	Pretreatment C	*T*_1_	*T*_2_	*T*_1_	*T*_2_
A1	B1	C1	−1	−1	50	5
A2	B2	C2	−1	−1	50	5
A3	B3	C3	−1	−1	50	5
A4	B4	C4	1	−1	70	5
A5	B5	C5	1	−1	70	5
A6	B6	C6	1	−1	70	5
A7	B7	C7	−1	1	50	10
A8	B8	C8	−1	1	50	10
A9	B9	C9	−1	1	50	10
A10	B10	C10	1	1	70	10
A11	B11	C11	1	1	70	10
A12	B12	C12	1	1	70	10
A13	B13	C13	−1.414	0	45.8579	7.5
A14	B14	C14	−1.414	0	45.8579	7.5
A15	B15	C15	−1.414	0	45.8579	7.5
A16	B16	C16	1.414	0	74.1421	7.5
A17	B17	C17	1.414	0	74.1421	7.5
A18	B18	C18	1.414	0	74.1421	7.5
A19	B19	C19	0	−1.414	60	3.9644
A20	B20	C20	0	−1.414	60	3.9644
A21	B21	C21	0	−1.414	60	3.9644
A22	B22	C22	0	1.414	60	11.0355
A23	B23	C23	0	1.414	60	11.0355
A24	B24	C24	0	1.414	60	11.0355
A25	B25	C25	0	0	60	7.5
A26	B26	C26	0	0	60	7.5
A27	B27	C27	0	0	60	7.5
A28	B28	C28	0	0	60	7.5
A29	B29	C29	0	0	60	7.5
A30	B30	C30	0	0	60	7.5
A31	B31	C31	0	0	60	7.5
A32	B32	C32	0	0	60	7.5
A33	B33	C33	0	0	60	7.5
A34	B34	C34	0	0	60	7.5
A35	B35	C35	0	0	60	7.5
A36	B36	C36	0	0	60	7.5
A37	B37	C37	0	0	60	7.5
A38	B38	C38	0	0	60	7.5
A39	B39	C39	0	0	60	7.5

**Table 3:** Design matrix and response value for *MC* and *WI* tests.

Sample	Response		Sample	Response		Sample	Response	
	*MC* (%)	*WI*		*MC* (%)	*WI*		*MC* (%)	*WI*
A1	13.21	83.17	B1	13.58	80.28	C1	9.51	90.98
A2	13.12	83.53	B2	13.78	79.86	C2	9.26	91.45
A3	12.82	83.06	B3	14.07	80.59	C3	9.88	91.18
A4	12.46	83.81	B4	14.03	80.78	C4	9.57	91.35
A5	12.75	83.36	B5	13.62	81.05	C5	9.21	91.82
A6	13.04	83.44	B6	13.56	80.38	C6	9.14	90.87
A7	12.33	82.68	B7	13.15	79.41	C7	9.18	90.04
A8	12.48	82.53	B8	13.78	79.78	C8	9.45	90.25
A9	12.87	82.15	B9	13.26	79.21	C9	9.52	90.14
A10	10.07	83.44	B10	10.64	80.55	C10	6.22	91.42
A11	10.13	83.65	B11	10.72	80.22	C11	6.51	91.57
A12	10.28	83.37	B12	10.56	80.78	C12	6.62	91.32
A13	13.23	82.13	B13	14.07	79.24	C13	9.58	90.22
A14	13.27	82.17	B14	13.94	79.18	C14	10.02	90.18
A15	13.39	82.15	B15	14.12	79.28	C15	9.65	90.13
A16	10.84	83.29	B16	11.62	80.57	C16	7.23	90.82
A17	11.13	83.36	B17	11.86	80.45	C17	7.61	91.46
A18	10.72	83.29	B18	11.82	80.45	C18	7.78	91.62
A19	13.32	84.05	B19	14.35	81.13	C19	9.88	92.25
A20	12.88	84.05	B20	14.42	81.27	C20	10.13	91.72
A21	12.86	84.14	B21	14.56	81.15	C21	9.96	92.07
A22	10.82	83.07	B22	11.42	80.22	C22	7.34	91.58
A23	11.12	83.18	B23	11.74	80.21	C23	7.52	91.62
A24	11.07	83.24	B24	11.66	80.24	C24	7.65	91.46
A25	11.15	80.56	B25	11.58	78.16	C25	7.45	88.87
A26	11.12	80.83	B26	11.85	77.58	C26	7.45	89.17
A27	11.18	80.62	B27	11.75	77.95	C27	8.06	88.72
A28	11.55	80.91	B28	11.52	77.92	C28	7.73	88.56
A29	11.07	81.14	B29	12.03	77.72	C29	7.65	88.58
A30	11.12	80.71	B30	11.72	77.67	C30	7.58	88.61
A31	11.26	80.48	B31	11.73	77.71	C31	8.16	89.15
A32	10.83	81.06	B32	11.52	77.76	C32	7.86	88.68
A33	11.34	81.20	B33	12.07	77.74	C33	7.72	88.70
A34	10.83	80.61	B34	11.54	77.62	C34	7.61	88.64
A35	10.84	80.76	B35	11.07	77.67	C35	8.03	88.61
A36	10.87	80.82	B36	11.87	77.81	C36	7.54	88.82
A37	10.91	81.18	B37	11.74	77.80	C37	7.56	89.12
A38	11.12	80.59	B38	11.63	78.24	C38	7.87	88.68
A39	11.14	80.61	B39	11.56	78.06	C39	7.94	88.94

**Table 4:** *MC* for different *T*_1_ and *T*_2_.

Source	Notation	Sum of Squares	Mean Square	Coefficient	Standard Error	*p*	*R* ^2^	*R*^2^ (adj)
Pretreatment A								
Constant				11.0887	0.0560	0.000	0.9624	0.9567
Temperature	*T*_1_	13.93	13.93	−0.7618	0.0443	0.000		
Time	*T*_2_	13.20	13.20	−0.7415	0.0443	0.000		
Temperature∗time	*T*_1_ *T_2_	3.31	3.31	−0.5250	0.0626	0.000		
Temperature∗temperature	*T*_1_ *T_1_	5.71	5.71	0.5230	0.0475	0.000		
Time∗time	*T*_2_ *T_2_	4.82	4.82	0.4805	0.0475	0.000		
Lack of fit		0.2347	0.0782			0.172		
Error		1.32	0.0440					
Total		41.30						
Pretreatment B								
Constant				11.6787	0.0572	0.000	0.9713	0.9669
Temperature	*T*_1_	13.72	13.72	−0.7562	0.0453	0.000		
Time	*T*_2_	21.22	21.22	−0.9402	0.0453	0.000		
Temperature∗time	*T*_1_ *T_2_	5.40	5.40	−0.6708	0.0640	0.000		
Temperature∗temperature	*T*_1_ *T_1_	7.41	7.41	0.5959	0.0485	0.000		
Time∗time	*T*_2_ *T_2_	8.98	8.98	0.6559	0.0485	0.000		
Lack of fit		0.1800	0.0600			0.310		
Error		1.44	0.0481					
Total		56.47						
Pretreatment C								
Constant				7.7473	0.0573	0.000	0.9648	0.9595
Temperature	*T*_1_	14.89	14.89	−0.7878	0.0453	0.000		
Time	*T*_2_	16.04	16.04	−0.8175	0.0453	0.000		
Temperature∗time	*T*_1_ *T_2_	5.43	5.43	−0.6725	0.0640	0.000		
Temperature∗temperature	*T*_1_ *T_1_	4.10	4.10	0.4430	0.0485	0.000		
Time∗time	*T*_2_ *T_2_	5.09	5.09	0.4938	0.04854	0.000		
Lack of fit		0.0955	0.0318			0.604		
Error		1.53	0.0509					
Total		46.12						

**Table 5:** *WI* for different *T*_1_ and *T*_2_.

Source	Notation	Sum of Squares	Mean Square	Coefficient	Standard Error	*p*	*R* ^2^	*R*^2^ (adj)
Pretreatment A								
Constant				80.8053	0.0529	0.000	0.9774	0.9739
Temperature	*T*_1_	3.29	3.29	0.3702	0.0418	0.000		
Time	*T*_2_	1.73	1.73	−0.2683	0.0418	0.000		
Temperature∗time	*T*_1_ *T_2_	0.4219	0.4219	0.1875	0.0592	0.003		
Temperature∗temperature	*T*_1_ *T_1_	19.42	19.42	0.9646	0.0449	0.000		
Time∗time	*T*_2_ *T_2_	41.47	41.47	1.4096	0.0449	0.000		
Lack of fit		0.1154	0.0385			0.449		
Error		1.27	0.0424					
Total		61.24						
Pretreatment B								
Constant				77.8273	0.0547	0.000	0.9772	0.9737
Temperature	*T*_1_	4.13	4.13	0.4150	0.0432	0.000		
Time	*T*_2_	2.08	2.08	−0.2942	0.0432	0.000		
Temperature∗time	*T*_1_ *T_2_	0.2324	0.2324	0.1392	0.0611	0.029		
Temperature∗temperature	*T*_1_ *T_1_	21.15	21.15	1.0068	0.0463	0.000		
Time∗time	*T*_2_ *T_2_	42.53	42.53	1.4276	0.0463	0.000		
Lack of fit		0.0798	0.0266			0.639		
Error		1.40	0.0466					
Total		64.75						
Pretreatment C								
Constant				88.79	0.0656	0.000	0.9657	0.9605
Temperature	*T*_1_	3.43	3.43	0.3782	0.0519	0.000		
Time	*T*_2_	0.9848	0.9848	−0.2026	0.0519	0.004		
Temperature∗time	*T*_1_ *T_2_	0.9919	0.9919	0.2875	0.0734	0.004		
Temperature∗temperature	*T*_1_ *T_1_	17.55	17.55	0.9171	0.0556	0.000		
Time∗time	*T*_2_ *T_2_	43.25	43.25	1.4396	0.0556	0.000		
Lack of fit		0.3597	0.1199			0.130		
Error		1.77	0.0591					
Total		62.08						

**Table 6:** Experiment Verification.

Sample	MC (%)			WI		
	Predicted	Actual	Relative Deviation (%)	Predicted	Actual	Relative Deviation (%)
Pretreatment A						
AV1	10.06	10.12	0.59	83.47	83.62	0.18
AV2	10.06	10.23	1.68	83.47	83.35	0.14
AV3	10.06	10.28	2.16	83.47	83.43	0.05
	Mean		1.48	Mean		0.12
Pretreatment B						
BV1	10.56	10.36	1.91	80.52	80.68	0.20
BV2	10.56	10.71	1.68	80.52	80.38	0.17
BV3	10.56	10.47	2.16	80.52	80.61	0.11
	Mean		1.48	Mean		0.16
Pretreatment C						
CV1	6.41	6.37	0.63	91.61	91.42	0.21
CV2	6.41	6.54	2.01	91.61	91.57	0.04
CV3	6.41	6.49	1.24	91.61	91.83	0.24
	Mean		1.29	Mean		0.16

## Data Availability

Data are contained within the article.
